# Waiver of Consent in a Trial Intervention Occurring at Birth—How Do Parents Feel?

**DOI:** 10.3389/fped.2017.00056

**Published:** 2017-03-21

**Authors:** Wade D. Rich, Anup C. Katheria

**Affiliations:** ^1^Neonatal Research Institute at Sharp Mary Birch Hospital for Women and Newborns, San Diego, CA, USA

**Keywords:** neonatal resuscitation, waiver of consent, delayed cord clamping, umbilical cord milking, parental response

## Abstract

**Background:**

We have previously demonstrated that it is difficult to obtain a representative subject sample when conducting a randomized controlled trial (RCT) at or near the time of birth and obtaining antenatal consent. Waiver of consent has been used in neonatal trials, but parents’ reactions to being enrolled in these trials have never been reported.

**Methods:**

The parents enrolled in a RCT involving a waiver of consent with a post-delivery discussion were asked to take part in a brief survey. The survey questions included the timing of when parents were informed about the study, and how they felt about their infants being included in the study.

**Results:**

Forty-nine parents completed the online survey. Sixty-nine percent (*n* = 34) remembered a physician discussing their premature baby with them prior to delivery. Thirty-four percent (17) indicated the physician had discussed participation in the study prior to delivery. Sixty-nine percent (34) indicated that they had a positive or strongly positive feeling about the studies impact on their baby’s health.

**Conclusion:**

Our study demonstrates that the majority of responding parents of infants who have actually participated in a RCT with a waiver of consent process had a positive response, a minority had a neutral response, and none had a slightly negative or highly negative response to participation in the study.

## Introduction

Informed consent is a process ensuring that subjects enrolled in a research trial are appropriately informed of the risks and benefits. Traditionally, consent is obtained directly from the subjects or their parents in the case of minors when they are in a condition to understand the information and to participate in the consent process. In trials involving newborns who will receive an intervention at or near the time of birth, the process requires approaching families before delivery (antenatal consent) or, at least in the United States, obtaining a waiver of consent from a local or regional institutional review board. In other places such as Canada, the United Kingdom, and Australia, it is possible to obtain a delayed/deferred consent, where the intervention is carried out without consent, and then after birth, the parents are approached to discuss the trial and get permission to use their infant’s data. Delayed consent has been used in neonatal trials, where the interventions being studied are both deemed low risk and/or standard of care or where antenatal consent would not have been practicable (1–6). We recently completed a trial comparing two different methods of providing placental transfusion to premature newborns (the PREMOD trial) for which a waiver of consent was obtained from the local IRB. The two interventions for the trial included delayed cord clamping until 45 s after birth and umbilical cord milking with the unclamped umbilical cord slowly squeezed or milked four times before the cord is clamped. The results of this trial have been previously reported (4, 5).

The PREMOD trial was conducted at two tertiary neonatal intensive care units in the United States; Sharp Mary Birch Hospital for Women and Newborns (SMBHWN) and Loma Linda University Medical Center (LLU). The trial was approved for waiver by each of the hospital’s Institutional Review Boards. Both interventions are standard practice and are considered to have minimal risk to the neonate. The study met the 45 CFR 46.116(c) criteria for delayed consent based on the inability to conduct the trial without a waiver and the minimal risk of either intervention. Pregnant women, dated by their earliest ultrasound or last menstrual period at <32 weeks of gestation, were identified and recruited from the labor and delivery and antepartum floors. When appropriate and feasible (typically when mothers were admitted for at least 6 h), antenatal consent was obtained. We have previously demonstrated that obtaining only antenatal consent would have potentially excluded the sickest children, including those who might benefit most from one or the other intervention (7, 8). Therefore, in cases, where antenatal consent could not be obtained, parents were notified of the intervention after the fact by the obstetrician or research team and were approached and given the opportunity to allow their baby’s data to be used. If consent was withheld, the baby would be removed from the study and the data would be destroyed. A short survey was developed to explore how parents involved in the PREMOD trial felt about the effect of the waivered consent process on their baby’s care. The proportion of emergently delivered infants in this trial was assessed to validate if there were a representative number of infants in a trial done with delayed consent.

## Materials and Methods

Subjects enrolled in the PREMOD trial were asked by members of the research team during routine follow-up phone calls if they would be willing to provide their email address so that the parents could participate in a blinded survey regarding their participation in the PREMOD study. The calls were part of the normal scheduling of their neurodevelopmental follow-up visits for the study. Parents then received an email asking them to participate in the current study regarding delayed consent, explaining that their answers were anonymous. The blinded survey was conducted through an online survey program (SurveyMonkey). Investigators had access to responses but did not know who was completing the surveys. The survey consisted of seven questions and was approved by the Sharp HealthCare Investigational Review Board (IRB). Prior to the IRB submission, the survey was revised and edited through the Sharp Mary Birch Hospital for Women and Newborns Parent Advisory Board. The Parent Advisory Board is a committee of parents of former premature infants who routinely review Neonatal Research Institute protocols to ensure that they are appropriately worded for parents, and the study protocols are acceptable trials from a parental perspective. The survey was distributed between March 2015 and March 2016. A brief description of the trial was included as an introduction to the survey, including what the interventions were, and that the protocols were performed using delayed consent.

## Results

Of the 150 infants who were enrolled in the PREMOD trial at our site, 98 parents provided email addresses for the survey. Forty-nine parents completed the survey (Table 1). Sixty-nine percent remembered a physician discussing their premature baby with them prior to delivery, 26% did not believe they had such a discussion and 4% did not remember. Thirty-four percent (17) indicated the physician had discussed participation in the study prior to delivery. Eighty percent (39) of surveys were completed by the mother.

**Table 1:** **Responses to questionnaire**.

Did a NICU doctor talk to you before delivery about the possibility of having a premature baby?	Yes (69.4%)	No (26.5%)	Don’t remember (4.0%)		
How did you first learn about your baby’s participation in this study?	Before^a^ (20.4%)	After^b^ (48.9%)	Both (14.2%)	Don’t Remember (16.3%)	
Likert-like questions	Strongly negative	Negative	Neutral	Positive	Strongly positive
Which of the following describes how you feel about your baby’s participation in this study?	0%	0%	28.6%	32.6%	38.8%
What impact do you feel participation in this study had on your infant’s health?	0%	0%	30.6%	40.8%	28.6%
I would consider participation in another trial in the future	0%	0%	30.6%	38.7%	30.6%
How is the person filling out this survey related to the infant in the study?	Father (18.3%)	Mother (79.6%)	Together (4.0%)		

*^a^Before—my doctor talked to me before the delivery*.

*^b^After—my doctor or a member of the team talked to my about the study after delivery*.

## Discussion

This trial attempts to determine how parents of infants enrolled in a trial with waiver of consent feel about the process. In a survey of researchers whose primary focus was neonatal resuscitation, Foglia found that an overwhelming majority agreed with the statement that “Enrolling subjects for DR studies without antenatal informed consent is an acceptable tradeoff between respect for persons for enrolled subjects and potential benefit for all sick newborns” (9). Some investigators have voiced concerns that a disproportionate number of vulnerable, disadvantaged families are involved in clinical research (10, 11). In our previous investigations, we found just the opposite for studies involving antenatal consent. The review of subjects’ time from admission to the hospital until delivery demonstrated that at least 15% (23/150) delivered within 6 h of admission to the hospital, making it unlikely antenatal consent would have been obtained. These mothers had few or no antenatal steroids and magnesium and were more likely to have limited prenatal care.

Waiver of consent has been used in neonatal trials, where the interventions being studied are both deemed low risk and/or standard of care. The question of how parents feel about waived or delayed consent has not been fully answered. Culbert et al. used sample scenarios in a well-educated population and found that parents were less comfortable with delayed or waived consent than with a more conventional approach (12). In an exploratory trial of opting out compared to conventional consent in a group of 44 infants, Rogers et al. found that no significant differences between the groups when asked if they had any concern that there were risks about which they had not been told (13). A survey by Burgess et al. found that the vast majority of parents were not comfortable with physicians making the decision to enroll their baby in a trial, but several of these trials compared interventions that would not have been considered to be low risk (14). The timing of the survey used in this study is unique. Unlike situations like that of Culbert and Davis, the parents have already experienced the waivered consent process of two presumably beneficial interventions and were asked for permission to use additional data.

In an editorial of the PREMOD trial upon which this questionnaire was based, Tarnow-Mordi et al. suggested that delayed consent is the most appropriate way to conduct future trials (15). There is debate as to whether a waiver or deferred consent is appropriate in neonatal trials particularly when an intervention is deemed *above* minimal risk (16). This concern highlights the key point as to which interventions would fall in the above minimal risk category. It was reassuring that there were no parents who had negative or strongly negative feelings about the study based on their responses to the survey. In a trial of antenatal consent using identical interventions of placental transfusion, the parents’ responses were also positive, but many parents did not remember being enrolled in the study due to the stressful period of delivery. Parents commented that re-approaching them after the intervention, as was done in this study, would be helpful (17). In our experience, the most important benefit of waiver of consent is the opportunity to spend significantly more time in a non-stressful environment (after the baby has delivered and has been stabilized) and review the study in an manner that allows the parent to understand and even recall being in the trial. The positive responses from the parents reflect this.

The limitations of this study are the small numbers, which are a function of this being a single center neonatal trial completed in 1 year, and the limited data set, which was chosen to improve return of the survey. Also, because the information in this trial depends on parental recall, there is the possibility that what they recall does not reflect what actually occurred.

## Conclusion

We have shown previously that without waiver of consent, the population studied is not representative of the entire population, thus diminishing the scientific validity and generalizability of the findings. Institutional Review Boards should accept the lack of a negative parental reaction as further evidence that waiver of consent is an acceptable option for neonatal trials. It may be also worth emphasizing the question of how valid antenatal consent really is given the mother’s state of mind. We found that the vast majority (70%) of responding parents of infants who actually participated in a randomized controlled trial involving waiver of consent had a positive feeling about the studies impact on their baby’s health. We believe that future neonatal trials should continue to use this type of consent for low risk/standard of care interventions. Future studies using waiver of consent should also continue to follow-up with families to help ensure that our results remain consistent.

## Author Contributions

WR conceptualized and designed the study, drafted the initial manuscript, and approved the final manuscript as submitted. AK reviewed and revised the manuscript and approved the final manuscript as submitted.

## Conflict of Interest Statement

The authors declare that the research was conducted in the absence of any commercial or financial relationships that could be construed as a potential conflict of interest.
